# Cryptogamic stem covers may contribute to nitrous oxide consumption by mature beech trees

**DOI:** 10.1038/s41598-017-13781-7

**Published:** 2017-10-16

**Authors:** Katerina Machacova, Martin Maier, Katerina Svobodova, Friederike Lang, Otmar Urban

**Affiliations:** 1 Global Change Research Institute CAS, Belidla 986/4a, 603 00 Brno, Czech Republic; 2 Chair of Soil Ecology, Albert-Ludwigs-University, Bertoldstrasse 17, 79098 Freiburg, Germany

## Abstract

Naturally produced by microbial processes in soil, nitrous oxide (N_2_O) is an important greenhouse gas contributing to climate change. Accordingly, there is a need to accurately quantify the capability of forest ecosystems to exchange N_2_O with the atmosphere. While N_2_O emissions from soils have been well studied, trees have so far been overlooked in N_2_O inventories. Here, we show that stems of mature beech trees (*Fagus sylvatica*) may act as a substantial sink of N_2_O from the atmosphere under conditions of soils consuming N_2_O. Consistent consumption of N_2_O by all stems investigated (ranging between −2.4 and −3.8 µg m^−2^ h^−1^) is a novel finding in contrast to current studies presenting trees as N_2_O emitters. To understand these fluxes, N_2_O exchange of photoautotrophic organisms associated with beech bark (lichens, mosses and algae) was quantified under laboratory conditions. All these organisms were net N_2_O sinks at full rehydration and temperature of 25 °C. The consumption rates were comparable to stem consumption rates measured under field conditions. Cryptogamic stem covers could be a relevant sink of N_2_O in European beech forests.

## Introduction

Nitrous oxide (N_2_O) is the dominant substance depleting stratospheric ozone and contributes importantly to global radiative forcing that consequently leads to changes in the Earth’s climate^1^. Global N_2_O emissions into the atmosphere are estimated to be in the range of 8.1–30.7 Tg yr^−1^ ref.^1^. About two-thirds of these emissions are estimated to originate from natural terrestrial sources particularly related to microbial processes in soils^2^. N_2_O is a facultative by-product of major microbiological nitrogen (N) cycling processes, including autotrophic and heterotrophic nitrification, heterotrophic nitrification combined with aerobic denitrification, nitrifier denitrification, anaerobic denitrification, anaerobic dissimilatory nitrate reduction to ammonium, and abiotic chemodenitrification. The denitrification processes are the only processes reducing N_2_O to N_2_ ref.^3–6^. The net N_2_O exchange at the soil–atmosphere interface is therefore a balance of N_2_O production and consumption processes as well as of N_2_O transport within the soil^7^.

Temperate forests are estimated to emit around 1.6 kg N_2_O ha^−1^ yr^−1^ into the atmosphere and thus constitute an important natural source of N_2_O^8^. Forest soils are supposed to be responsible for the majority of emitted N_2_O, although net N_2_O uptake also has been reported^6,9,10^. In any case, the factors regulating N_2_O uptake by soil are not well understood^6^. To date, N_2_O fluxes between forests and the atmosphere have been calculated only based on N_2_O exchange at the soil–atmosphere interface. Plants have been shown to transport soil-derived N_2_O to the stems and leaves and emit it to the atmosphere^11–14^, and even to produce N_2_O during N assimilation processes^15,16^. However, trees have widely been overlooked as possible N_2_O emitters or have been presumed to play only a negligible role in forest ecosystems’ N_2_O exchange.

A few studies have revealed that trees can be important sources of N_2_O. This capability is found among various tree species of boreal and temperate zones, including conifers *Pinus sylvestris* and *Picea abies* as well as deciduous *Betula pendula*, *B. pubescens*, *Alnus glutinosa*, *Fraxinus angustifolia*, *Fagus sylvatica*, and poplar (*Populus*) hybrids^11–14,17–19^. However, these investigations have mostly been conducted using seedlings under artificial laboratory conditions of high fertilization or flooding to increase soil production of N_2_O. Reported N_2_O emissions by plants are thus unnaturally high and reflect plant-mediated transport of N_2_O from soil to the atmosphere^11–13^. Such transport is assumed to occur either via special gas transport tissue (aerenchyma system) in wetland tree species^11,13^ or in the liquid phase by the transpiration stream in non-aerenchymous species^20^.

To fully understand and accurately quantify natural terrestrial sources of N_2_O, all potential pathways must be taken into account. There is an urgent need, therefore, to investigate the capability of mature trees to exchange N_2_O in their natural growth environments. Moreover, the mechanisms responsible for N_2_O fluxes have to be clarified. In contrast to crop species^21^, there still remain numerous open questions related to the N_2_O exchange of trees and forest ecosystems. Among others, it is unclear how trees behave under natural conditions, and particularly when growing on soils with low N_2_O production. Moreover, until now the role of cryptogamic plant covers (i.e. photoautotrophic organisms such as cyanobacteria, algae, fungi, lichens and bryophytes)^22,23^ in tree N_2_O exchange is completely under-investigated, although the relevance of cryptogams for the N cycle of forest ecosystems was stressed in literature^24^.

Accordingly, the objective of our case study was to quantify natural N_2_O fluxes in mature European beech trees (*Fagus sylvatica*) representing native and widely distributed deciduous tree species in temperate forests of Central Europe. Field measuring campaigns were conducted during June and July 2015 at two mountain forest sites in the Czech Republic (Stitna, White Carpathians) and Germany (Conventwald, Black Forest) characterized by predominant soil N_2_O uptake. N_2_O exchange capacity of photoautotrophic organisms associated with beech bark was further investigated under laboratory conditions.

## Results and Discussion

**Figure 1:**
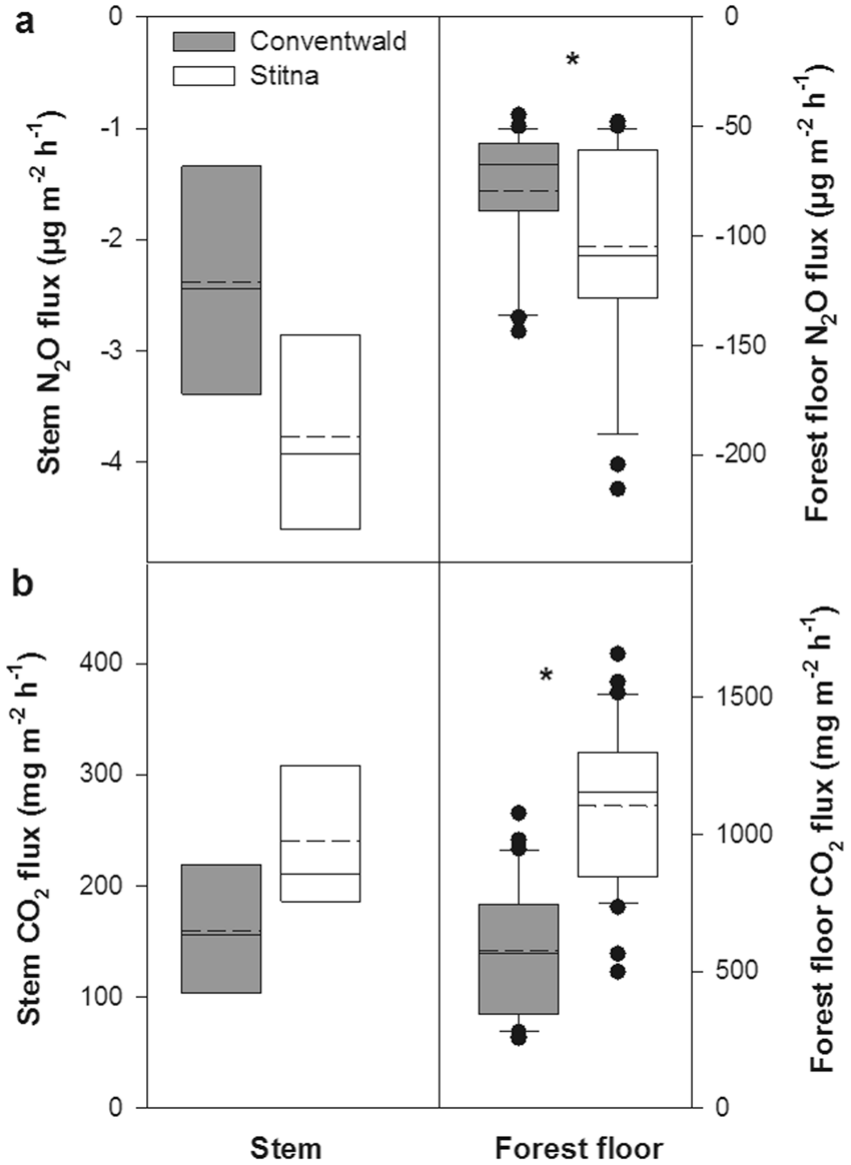
N_2_O (**a**) and CO_2_ (**b**) fluxes at beech stem and forest floor level in two beech forest stands (Conventwald, Black Forest; Stitna, White Carpathians) measured during June–July 2015. Negative fluxes indicate N_2_O uptake/consumption. Fluxes are expressed per m^2^ of stem or soil surface area, respectively. Stem fluxes were determined for five trees per forest stand (n = 5) at three stem heights with 3–4 measurement repetitions per each height and tree. Stem fluxes from different tree heights are presented together as they did not significantly vary with the tree height. Forest floor fluxes were quantified from 23 (n = 23) and 31 (n = 31) positions randomly distributed within the studied forest stands at Conventwald and Stitna, respectively. Fluxes are expressed as medians (solid lines) and means (broken lines). Boundaries within the boxes indicate 25th and 75th percentiles, and the whiskers 10th and 90th percentiles. Dots mark outliers. Statistically significant differences at *p* < 0.05 are indicated by asterisk.

Although the forest floor at both forest sites was a strong sink for N_2_O, the sink at Stitna (−105 µg N_2_O m^−2^ soil surface area h^−1^) was larger than that at Conventwald (−79 µg N_2_O m^−2^ h^−1^; Fig. 1a, Supplementary Fig. S1). Lower consumption rates at the Conventwald site were associated with lower CO_2_ efflux from the forest floor as compared to the Stitna site (Fig. 1b, Supplementary Figs S1,2). The difference in N_2_O and CO_2_ exchange rates between the sites might be related to soil temperature being lower in Conventwald than in Stitna during the measurement campaigns (at 0.3 m depth, 9.6 °C and 15.2 °C, respectively). The soil N_2_O concentration profiles revealed no clear production or consumption pattern in soils (Supplementary Fig. S3). Soils are regarded as N_2_O source, because N_2_O emissions are usually greater than N_2_O uptake^6^. While there exists a good conceptual understanding of the factors regulating N_2_O emissions and the processes involved^7^, there is only little knowledge about N_2_O consumption in soils^6^. Even though low mineral N and large soil water contents connected with low oxygen content have been reported in particular to favour N_2_O consumption, the fact that soil net N_2_O uptake has been measured also under different conditions complicates the identification of conditions suitable for such uptake^6^. The CO_2_ concentrations in soil profiles at both sites indicated aerobic conditions in the soil (Supplementary Fig. S4). Nevertheless, soil analysis showed a redoximorphic colour pattern at Stitna indicative of temporal and local anoxic conditions. Indications for reducing conditions were rarely observed at Conventwald. Yet oxygen depleted zones in the centre of aggregates, specific micro-sites in soils, were found^25^. These conditions are assumed to further contribute to anaerobic denitrification and heterotrophic nitrification combined with aerobic denitrification as the possible processes responsible for reducing N_2_O to N_2_ ref.^4–6^. It has also been shown, however, that nitrifiers can consume N_2_O in nitrifier denitrification^26^.

Stems of all mature beech trees studied were net sinks of N_2_O from the atmosphere (Fig. 1a, Supplementary Fig. S1). Even though the consumption rates at Stitna (−3.8 µg N_2_O m^−2^ stem surface area h^−1^) were higher than at Conventwald (−2.4 µg N_2_O m^−2^ h^−1^, Fig. 1a), the consumption rates of the majority of trees are in the same order of magnitude. Moreover, no significant changes (*p* > 0.05) in N_2_O consumption rates in the buttress roots area and with respect to stem height above the soil were observed (data not presented). Low N_2_O consumption rates by beech stems were associated with low respiratory CO_2_ efflux indicating a low physiological activity (Fig. 1, Supplementary Figs S1,2). To the best of our knowledge, such consistent consumption of N_2_O by tree stems constitutes a unique finding, as the limited number of current studies report trees only as N_2_O sources. Strong N_2_O emissions have been found, however, only under conditions of high N fertilization and/or N_2_O fumigation of roots^12,14,17^ or with short-term flooding treatment^11,13^. These manipulations stimulate N turnover processes and increase N_2_O production in soils. Enhanced N_2_O is consequently absorbed by tree roots, is transported along the transpiration stream to stems and/or leaves, and is then emitted to the atmosphere^12,13^. Even though the aforementioned studies show that trees, including mature beech trees^14^, have the ability to emit N_2_O, the N_2_O fluxes thus obtained cannot be used in estimating natural capability of trees to exchange N_2_O. Studies conducted under natural forest conditions have shown only very low N_2_O emissions close to, or even less than, the detection limit in various tree species^14,18,19^.

The scaled-up rates of N_2_O consumption by beech stems (−35.2 and −12.1 mg N_2_O ha^−1^ ground area h^−1^ at Stitna and Conventwald, Supplementary Fig. S1) contributed 3.4% to the forest floor N_2_O uptake at the Stitna (−1046 mg N_2_O ha^−1^ h^−1^) and 1.5% at the Conventwald (−793 mg N_2_O ha^−1^ h^−1^) stands. The upscaling procedure, based on mean tree constitution and tree density per hectare estimated for Stitna and Conventwald (see Methods), assumed constant consumption rates within the stem profile.

The bark of all studied trees at both forest sites was largely covered by cryptogamic plants amounting to as much as 40% of total stem area up to 5 m stem height. The cryptogam communities consisted particularly of lichens and fungi (*Graphis scripta* with identified *Cryptosporiopsis* sp. and *Pezicula* sp. fungi; mixture of *Graphis scripta* and *Lecanora sp*.; *Pseudevernia furfuracea;* non-determinable lichen), mosses (*Hypnum cupressiforme*), and red algae. Accordingly, we investigated whether these organisms can be involved in the observed N_2_O uptake.

**Figure 2:**
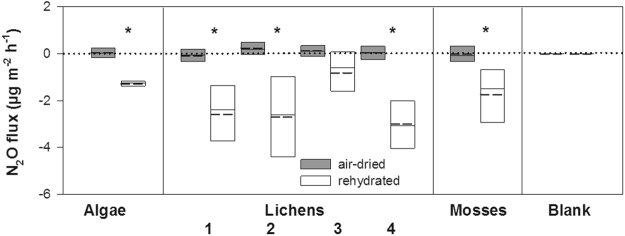
N_2_O fluxes in cryptogams sampled from beech bark in Conventwald, Black Forest. Fluxes are expressed per unit of stem projected area. The plotted results are medians (solid lines) and means (broken lines) of three measurement repetitions in air-dried and rehydrated cryptogams (n = 3). The following samples were investigated. Algae – red alga/Rhodophyta. Lichens: sample 1 – lichen *Graphis scripta* with identified *Cryptosporiopsis* sp. and *Pezicula* sp. fungi; sample 2 – mixture of lichens *Graphis scripta* and *Lecanora* sp.; sample 3 – lichen (undetermined); sample 4 – lichen *Pseudevernia furfuracea* (sampled from beech branch). Mosses – *Hypnum cupressiforme*. Blank samples (represented by dry and wet filter papers) were also analysed to exclude the possibility that N_2_O was diluted in water or leaked from the system (see Methods). For box plot description, see Fig. 1. Statistically significant differences in fluxes between air-dried and rehydrated organisms at *p* < 0.05 are indicated by asterisk.

Incubation experiments consistently revealed that all cryptogams tested were net N_2_O sinks under the conditions of full rehydration and room temperature amounting to −0.016 µg N_2_O g^−1^ dry weight h^−1^ (Fig. 2, Supplementary Fig. S5). Our test measurements confirmed that the observed N_2_O fluxes cannot be explained by N_2_O dilution in water (see blank samples in Fig. 2). The measurements were done at low light intensities below compensation irradiance (10–15 µmol m^−2^ s^−1^). Respiration processes thus dominated over photosynthesis^27^ and led to a permanent increase of CO_2_ concentration within close gas-exchange system (Supplementary Fig. S6). Linear changes in N_2_O and CO_2_ concentrations during first 210 minutes however document that the metabolic processes were not inhibited by high CO_2_ concentration as it could be observed under high light intensities when the phosphorylated intermediates form^28^.

Meanwhile, the activity of air-dried cryptogams was negligible (Fig. 2, Supplementary Figs S5,7,8). Air-dried cryptogams showed a very low CO_2_ exchange (10 µg CO_2_ g^−1^ h^−1^), whereas rehydration of the organisms led to rapid increase of their CO_2_ exchange (545 µg CO_2_ g^−1^ h^−1^, Supplementary Figs S7,8) and also of N_2_O consumption. These findings show general agreement that the physiological activity of cryptogams depends on water availability^23^. Moreover, we have found that high N_2_O consumption rates by cryptogams are associated with high CO_2_ emission rates (*p* < 0.001; Supplementary Fig. S9). Therefore, the role of cryptogamic bark covers in the N_2_O exchange of trees and forest ecosystems seems closely related to local climatic and meteorological conditions and should be studied in more detail in future, and especially so in relation to global climate scenarios.

We roughly estimated how much N_2_O is taken up by the active rehydrated cryptogams per stem area unit when fully covered with these organisms (the bark in stem chambers was as much as 90% covered with these organisms). The estimated N_2_O consumption rates per unit area (Fig. 2) were on the same order of magnitude as were stem consumption rates measured under the field conditions (Fig. 1). Specifically, it was −1.3, −2.0, and −1.8 µg N_2_O m^−2^ h^−1^ for algae, lichens, and mosses, respectively. Therefore, it seems that the bark vegetation is at least co-responsible for the observed unique consumption of N_2_O by beech trees. The contribution of cryptogams to N_2_O consumption can increase with rising water content (e.g. due to high air humidity or rainwater’s retention in the crotches of branches).

Our finding of N_2_O consumption is in contrast to those of studies^23,29^ presenting green algae and cryptogamic ground and plant covers as sources of N_2_O (mean emissions of 7.9 ng N_2_O g^−1^ h^−1^ ref.^23^). N_2_O is thought to be produced during an aerobic NO_3_^−^ assimilation, similar to proposed production in plants^15,16,23,29,30^. The difference in sink versus source character of cryptogamic stem covers may result from the fact that the cryptogams showing N_2_O emissions were investigated under dark conditions^23^, whereas our incubation experiments were performed under low light intensity of 10–15 µmol m^−2^ s^−1^ corresponding to low light conditions of forest understories. Moreover, moss-dominated communities in polar deserts can serve also as sinks for N_2_O under light conditions^31^. The authors hypothesize that the Bryophytes lack roots needed for N_2_O absorption from soil water. Cryptogamic plant covers with associated cyanobacterial communities are estimated to fix N_2_ up to 49 Tg yr^−1^ from the atmosphere, which amount corresponds to nearly half of the global terrestrial biological N_2_ fixation^22^. However, there is no known association of our sampled photoautotrophic organisms with N_2_-fixing cyanobacteria^32^. We therefore hypothesize that the absorbed N_2_O can serve as an additional source of N for the cryptogams lacking cyanobacteria. Absorbed N_2_O might also be partly re-emitted back into the atmosphere^23^, the rates of consumption might exceed the emission rates and thus result in net N_2_O consumption. Absorbed N_2_O might perhaps be partly converted to NO_3_^−^, similarly as is nitric oxide (NO)^33^, and then leached by rain. Absorbed N_2_O might be also reduced to N_2_ by denitrification microorganisms^6^ associated with cryptogams and N_2_ can be re-emitted into the atmosphere. Further detailed research is needed to understand the mechanisms behind the N_2_O uptake in cryptogams and trees, and the fate of the absorbed N_2_O molecules.

This study shows for the first time that not only soil but also mature trees can constitute an important sink for N_2_O under natural field conditions, thus increasing the sink capacity for N_2_O of forest ecosystems overall. We have found that N_2_O consumption rates are directly proportional to respiratory CO_2_ fluxes indicating overall physiological activity of trees and microbial communities. This paper also underlines the heretofore unknown role of cryptogamic plant covers in N_2_O consumption, which activity dramatically increases under wet conditions. The results of this case study need to be verified by a larger future project directed to studying spatial and seasonal variability in N_2_O exchange of beech trees while including also fluxes in leaves and branches, as well as to understanding the role of cryptogams in the beech N_2_O exchange under different climatic conditions.

## Methods

### Sites description

Measurements were conducted at two mountain forest sites in Central Europe. The Conventwald research site (48.02°N, 7.96°E; elevation 840 m a.s.l.; Black Forest, Germany) is in a mixed forest stand (density of 186 trees ha^−1^) with prevailing 135-year-old beech trees, followed by Silver fir (*Abies alba*) and Norway spruce (*Picea abies*)^34^. Average beech height and stem diameter at breast height were 30 m and 0.58 m, respectively. Mean annual temperature and precipitation are 8.5 °C and 1330 mm, respectively^35^.

The Stitna research site (49.02°N, 17.58°E; elevation 550 m a.s.l.; White Carpathians, Czech Republic) is a 115 year-old beech monoculture (density of 283 trees ha^−1^) with average tree height of 33 m and mean stem diameter at breast height of 0.60 m. Mean annual temperature and total annual precipitation are 7.5 °C and 800 mm, respectively^36^. The soil type at both stands is Cambisol (Conventwald: Hyperdistric Skeletic Cambisol; Stitna: Eutric (Stagnic) Cambisol)^37,38^. The soil texture at Conventwald is sandy loam with 30–60% coarse soil fraction and at Stitna loam with no coarse soil fraction. Soil pH was 3.5 for Conventwald and 7.0 for Stitna^38^. See Maier *et al*. (2017)^38^ for detailed soil characteristics of both sites.

During the measuring campaign (June–July 2015), mean air temperatures at Conventwald and Stitna were 16.8 °C and 19.2 °C, soil volumetric water content (0.3 m depth) 22% and 26%, and soil temperatures (0.3 m depth) 9.6 °C and 15.2 °C, respectively.

### Stem fluxes of N_2_O and CO_2_

Stem N_2_O and CO_2_ fluxes were determined in five representative beech trees of average height and stem diameter at each site (n = 5). The stem flux measurements were repeated three to four times during the measuring campaign. All fluxes were measured between 10:00 and 16:00 on sunny days to prevent possible variation caused by diurnal cycle. Stem fluxes were simultaneously measured at three stem heights (0.4, 1.2 and 2.0 m above the ground) at each tree using a static chamber system. Three to four large (internal volume of 2.1 dm^3^) or small (internal volume of 0.9 dm^3^) rectangular stem chambers were installed at different sides of the stem for each stem height in order to representatively cover the stem circumferential surface area. The large chambers covered an area of 0.0183 m^2^ each, the small ones of 0.0084 m^2^ each. The chambers at one stem height were interconnected in series using polyurethane tubes. A constant flow rate and mixing of the air inside the system were provided by a DP0140/12 V pump (Nitto Kohki, Tokyo, Japan). The chambers were made from transparent plastic storage containers with removable airtight lids (Lock & Lock, Anaheim, CA, USA). The bottom part of each container was cut out and glued to a 2 cm thick neoprene frame. The chambers were sealed with silicone to the carefully smoothed bark surface several days before the measurement campaigns and tested for leakage. All chambers were left open between measuring campaigns.

During the measurements, seven gas samples (20 ml) were taken via a septum at 0-30-60-90-130-170-210 min after chamber system closure when the linear changes in N_2_O and CO_2_ concentrations were observed. Changes in chamber pressure caused by gas sample uptake were tested and compensated by an insertion of empty needle to the system resulting in a negligible under-pressure of approx. 10 mbar for less than 5 seconds. The gas samples were stored in pre-evacuated gas-tight glass vials (Labco, Ceredigion, UK) at 7 °C until analysis. A Tracera gas chromatograph (Shimadzu Corporation, Kyoto, Japan) equipped with a barrier discharge ionization detector operated at 250 °C with helium as carrier gas was used to determine N_2_O and CO_2_ concentrations. A coupled ShinCarbon ST micro column (Restek, Bellefonte, PA, USA) with a length of 2 m and internal diameter of 1 mm and fused silica capillary (Restek, USA) of internal diameter 0.53 mm were used for gas separation. The oven temperature began at 70 °C for 14 min followed by its increase to 200 °C for 1.5 min. The gas samples were automatically injected by a GX-271 autosampler (Gilson, Middleton, Wisconsin, USA). The N_2_O and CO_2_ peaks were identified using LabSolutions software (Shimadzu Corporation, Japan). N_2_O and CO_2_ concentrations were calculated based on a four-concentration calibration curve (0.29, 0.43, 0.56, 0.70 µmol N_2_O mol^−1^; 400, 667, 933, 1200 µmol CO_2_ mol^−1^).

The stem fluxes were calculated linearly based on the gas concentration changes over time (for examples see Supplementary Fig. S6)^19^ and expressed per m^2^ of stem surface area. The fluxes were further scaled up to per-hectare values for beech forest. Extrapolation was based on the estimated mean stem surface area (33.0 and 27.3 m^2^ per tree in Stitna and Conventwald, respectively) and tree density (283 and 186 trees ha^−1^ in Stitna and Conventwald, respectively).

### Forest floor fluxes of N_2_O and CO_2_

The fluxes between forest floor and atmosphere were measured simultaneously with stem fluxes at 23 (n = 23, Conventwald) and 31 (n = 31, Stitna) positions randomly spread over the studied forest sites to cover the spatial heterogeneity of the soil N_2_O and CO_2_ fluxes.

The manual cylindrical soil chambers constructed of polyvinyl chloride with internal diameter of 0.17 m and height of 0.25 m ref.^39^ were installed one day before the measurements^38^. The chambers were connected with two gas analysers via polyurethane tubing fixed in the chamber lids, thus forming a closed flow-through system. Changes in N_2_O concentration were detected by a Photoacoustic Innova Field Gasmonitor (LumaSense Technologies, Ballerup, Denmark), changes in CO_2_ by a Greenhouse Gas analyzer (Ultraportable, Los Gatos Research, San Jose, CA, USA). The moisture content of the sampled air was conditioned using a dew point controller set to 8 °C. This stabilization of the H_2_O concentration allowed for high-precision N_2_O measurement with the LumaSense device. The fluxes were calculated based on the linear least square fits of time series of N_2_O and CO_2_ concentrations measured in the headspace.

### Soil N_2_O and CO_2_ profile

To investigate production and consumption of N_2_O and CO_2_ in the soil, soil gas profiles were determined using a multi-level soil gas sampler (+0.03, 0, −0.05, −0.1, −0.2, −0.3, −0.4 m sampling depth with 0 m as interface between mineral soil and humus layer)^40^ installed at distances of 1, 2 and 4 m from the tree stem. Two beech trees out of the five selected for stem measurements were chosen per site for the soil gas profiles. The soil gas samplers were connected to the two gas analysers via a polyurethane tubing system to determine the N_2_O and CO_2_ concentrations in the soil profiles. The different sampling depths were consecutively measured.

### Laboratory measurements of cryptogams

Photoautotrophic organisms (lichens, mosses and algae) forming cryptogamic covers of beech stems were collected in Conventwald for further analyses. To prevent any disruption of the bark-microcosm within the stem chambers, the cryptogams were sampled from the bark outside these chambers. After collection, samples were air-dried and stored in paper bags. In total, samples of six communities were collected: 1) Algae – red alga/Rhodophyta, 2) lichen *Graphis scripta* with identified *Cryptosporiopsis* sp. and *Pezicula* sp. fungi, 3) mixture of lichens *Graphis scripta* and *Lecanora* sp., 4) lichen (undetermined), 5) lichen *Pseudevernia furfuracea* (sampled from beech branch), and 6) moss *Hypnum cupressiforme*.

Gas exchange measurements were performed under constant laboratory conditions, i.e. air temperature of 25 °C and light intensity of 10–15 µmol m^−2^ s^−1^ corresponding to low light conditions of forest understories. Measurements of air-dried and fully rehydrated cryptogams were performed to investigate the effect of physiological activity on their N_2_O fluxes. To activate the air-dried organisms, samples were sprinkled with a mixture of rain and distilled water until full rehydration was reached. To ensure activation of the rehydrated cryptogams, the measurements were started ca 2.5 h after the addition of water.

For gas exchange (N_2_O and CO_2_) measurements, the cryptogams were placed in 600 ml plastic gas-tight containers (Lock & Lock, USA) with installed septa for gas sampling. The air (20 ml) was sampled at 0-60-130-220-320-420 min after container closure (air-dried cryptogams) or at 0-30-60-100-150-210 min (rehydrated cryptogams) and stored in pre-evacuated gas-tight glass vials at 7 °C. The possible pressure changes in the system caused by gas sample uptake were compensated. The concentrations of N_2_O and CO_2_ in air samples were assessed by gas chromatography as described above. The N_2_O and CO_2_ fluxes between cryptogams and the atmosphere were calculated as the slope of the linear regression indicating the change of concentration with time (see Supplementary Fig. S6)^19^ and expressed per unit of dry weight of cryptogams and unit of stem surface area fully covered with cryptogams. The field measurement of the stem area covered with sampled cryptogams of known dry weight allowed quantification of N_2_O and CO_2_ flux rates by cryptogams related to stem surface area unit. In addition, three types of blank samples were analysed to exclude the possibility that N_2_O would be diluted in water or leak from the system. These blanks consisted of i) containers filled with 8 layers of filter paper saturated with distilled water, ii) containers filled with the same amount of dry filter paper, and iii) empty containers.

### Statistics

The sets of fluxes were tested for normal distribution (Shapiro–Wilk test) and equality of variances in different subpopulations. A *t*-test was applied for the normally distributed data and a non-parametric Mann–Whitney rank-sum test for the non-normally distributed data and/or data with unequal variances. The n values for statistical analyses are stated in the figure legends. Statistical significance for all tests was defined at *p* < 0.05. The relationships between N_2_O and CO_2_ fluxes were tested by linear regression at *p* < 0.001. The statistics were run with SigmaPlot 11.0 (Systat Software, San Jose, California, USA).

### Data Availability

The datasets generated and analysed during this study are available from the authors on reasonable request.

## Electronic supplementary material

Supplementary Figures
