# Supplementary material for: Cryptogamic stem covers may contribute to nitrous oxide consumption by mature beech trees
Source: Sci Rep. 2017 Oct 16;7:13243. doi: 10.1038/s41598-017-13781-7 (PMC5643534; doi:10.1038/s41598-017-13781-7)
Supplement: Supplementary file 1 — Supplementary Figures [file 41598_2017_13781_MOESM1_ESM.pdf]

Supplementary Information

Cryptogamic stem covers may contribute to nitrous oxide consumption by mature beech trees

Katerina Machacova[1,*], Martin Maier[2], Katerina Svobodova[1], Friederike Lang[2] & Otmar Urban[1]

[1]Global Change Research Institute CAS, Belidla 986/4a, 603 00 Brno, Czech Republic

[2]Chair of Soil Ecology, Albert-Ludwigs-University, Bertoldstrasse 17, 79098 Freiburg, Germany

*corresponding author, machacova.k@czechglobe.cz

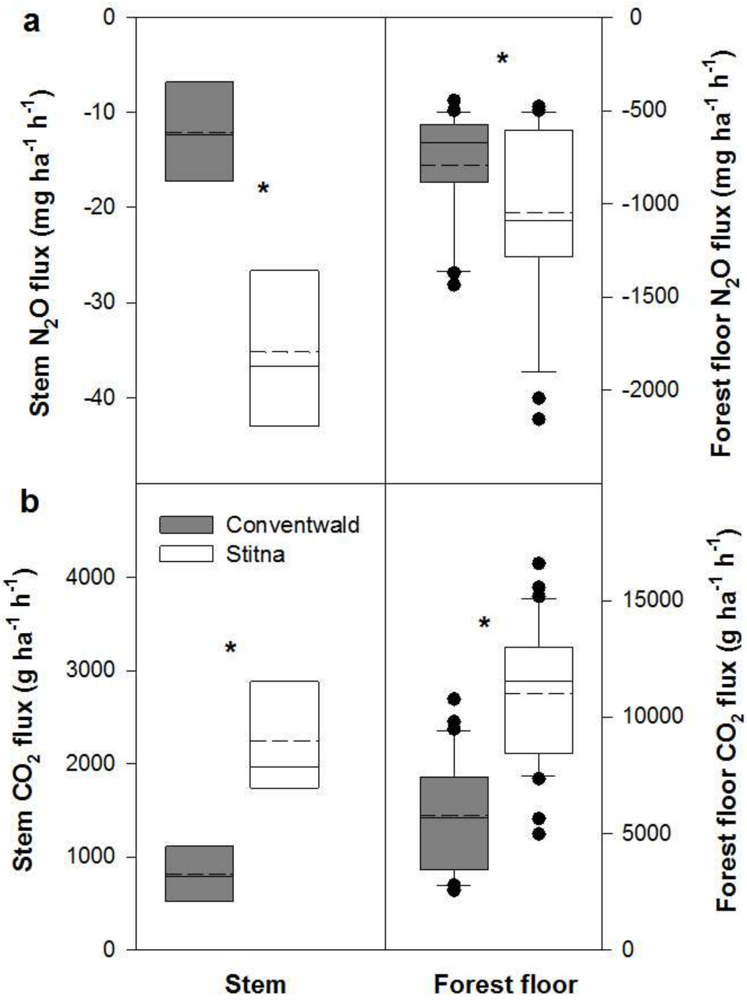

Supplementary Figure S1. N_2O (a) and CO_2 (b) fluxes at beech stem and forest floor level scaled up per unit ground area of two beech forest stands (Conventwald, Black Forest; Stitna, White Carpathians), measured during June–July 2015. For further information, see Fig. 1.

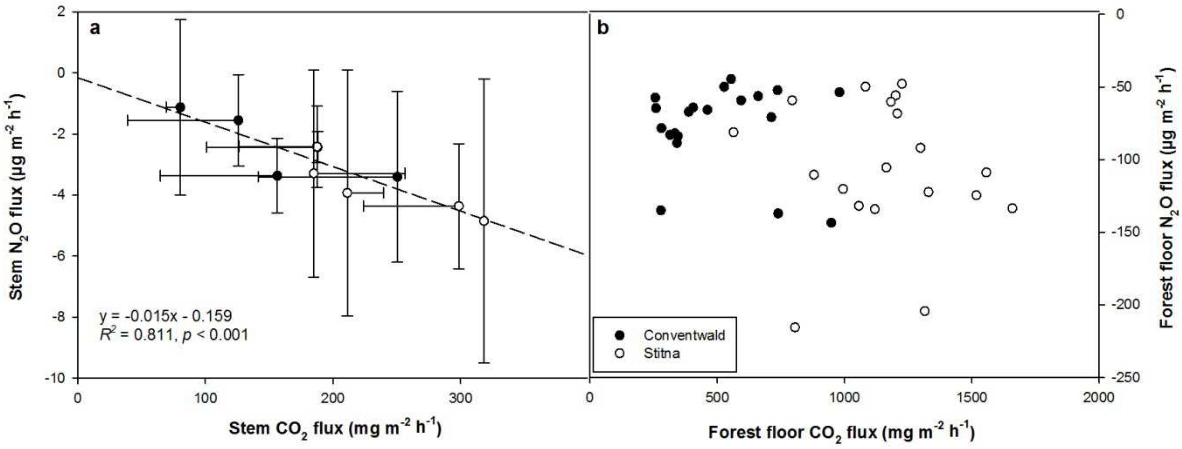

Supplementary Figure S2. N_2O versus CO_2 fluxes at beech stem (a) and forest floor (b) level in two beech forest stands (Conventwald, Black Forest; Stitna, White Carpathians). For further information about flux determination, see Fig. 1.

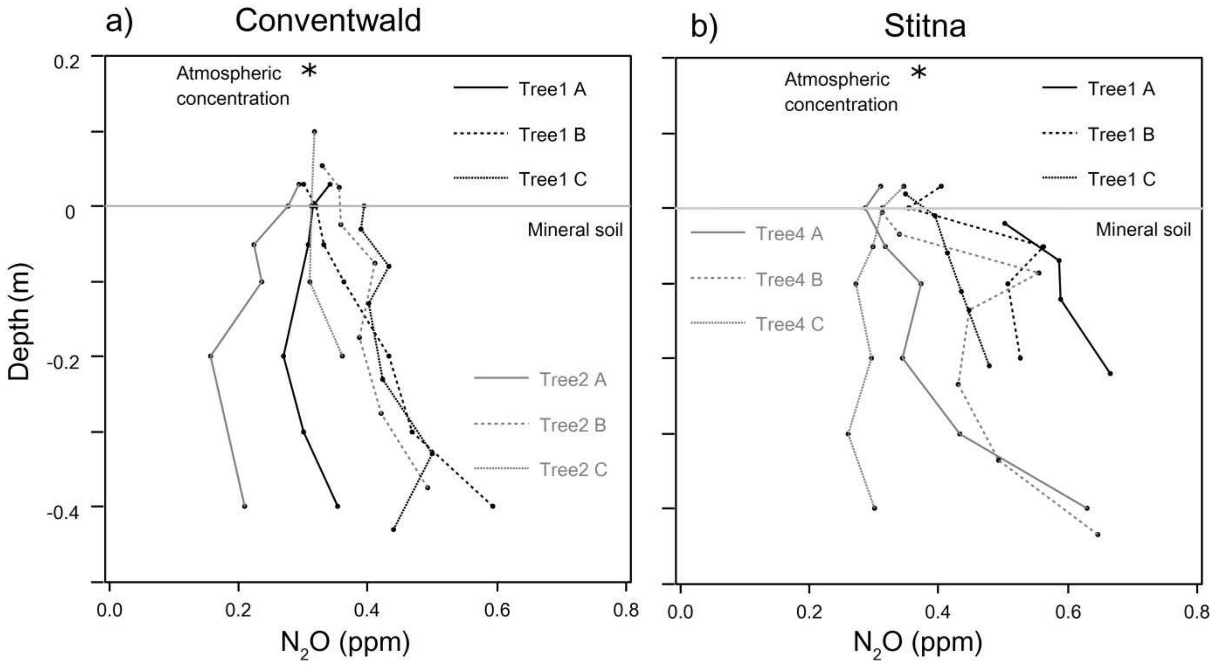

Supplementary Figure S3. Soil N_2O profiles close to two beech trees in Conventwald (a) and two trees in Stitna (b). Profiles A, B and C lie on a straight line (transect) from stems towards the outer rim of the crown (profile A next to tree stem).

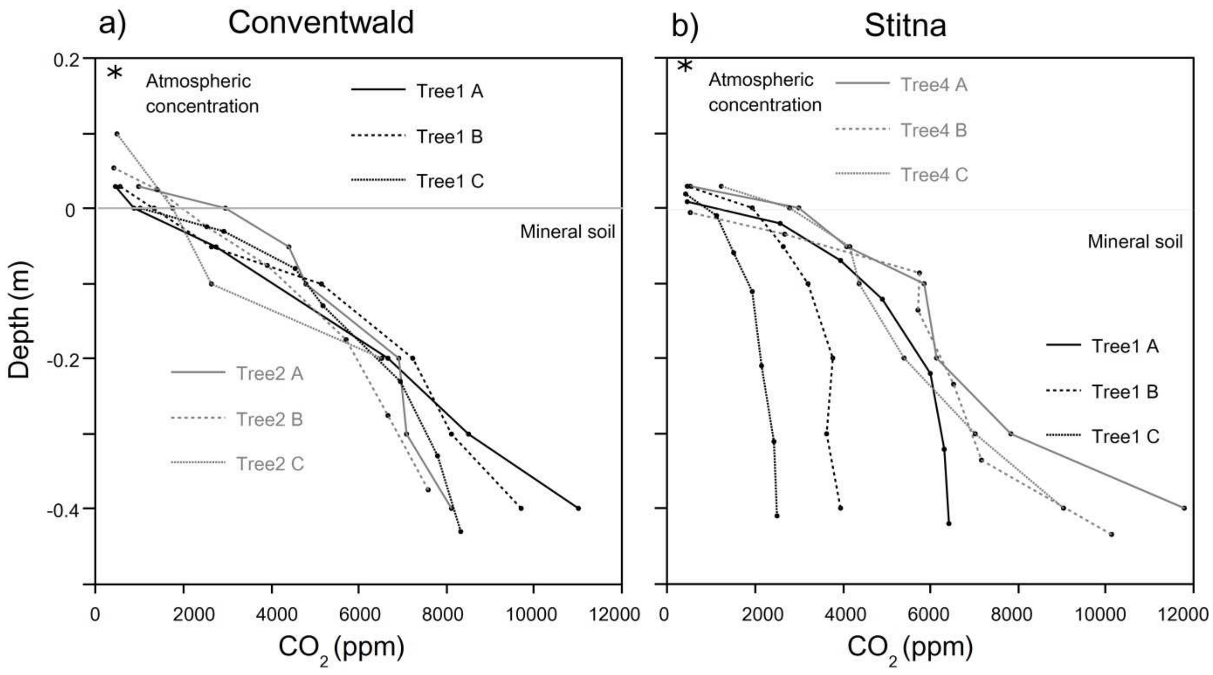

Supplementary Figure S4. Soil CO_2 profiles close to two beech trees in Conventwald (a) and two trees in Stitna (b). Profiles A, B and C lie on a straight line (transect) from stems towards the outer rim of the crown (profile A next to tree stem).

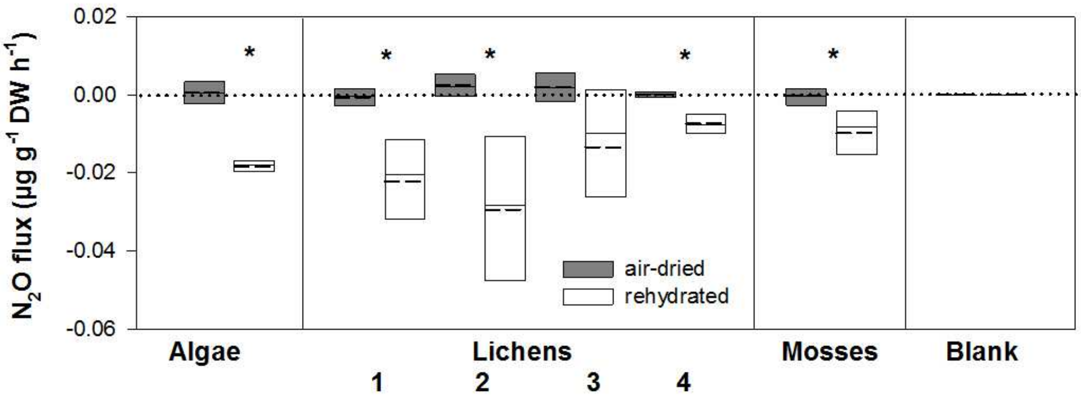

Supplementary Figure S5. N_2O fluxes in cryptogams sampled from beech bark in Conventwald, Black Forest. The fluxes are expressed per cryptogam dry weight (DW) unit. For further information regarding flux determination, taxonomic composition of cryptogams and box plot description, see Fig. 2.

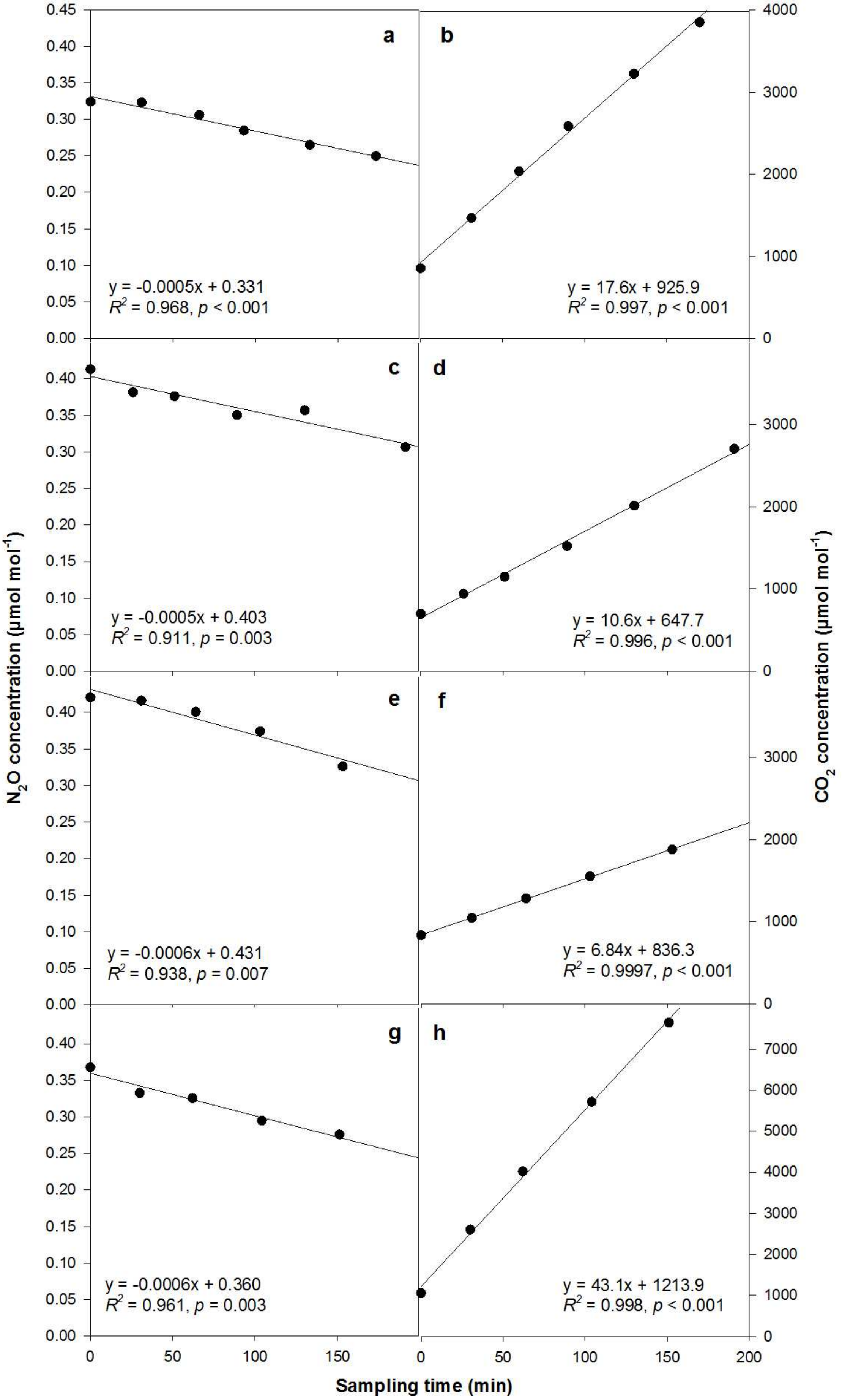
a
b
c
d
e
f
g
h
y = -0.0005x + 0.331
R2 = 0.968, p < 0.001
y = 17.6x + 925.9
R2 = 0.997, p < 0.001
y = -0.0005x + 0.403
R2 = 0.911, p = 0.003
y = 10.6x + 647.7
R2 = 0.996, p < 0.001
y = -0.0006x + 0.431
R2 = 0.938, p = 0.007
y = 6.84x + 836.3
R2 = 0.9997, p < 0.001
y = -0.0006x + 0.360
R2 = 0.961, p = 0.003
y = 43.1x + 1213.9
R2 = 0.998, p < 0.001
N2O concentration (µmol mol-1)
CO2 concentration (µmol mol-1)
Sampling time (min)

Supplementary Figure S6. Examples of N_2O (on the left) and CO_2 (on the right) concentration changes over time in headspace of stem chambers (a,b), and containers containing red alga/Rhodophyta (c,d), lichens (e,f) and mosses *Hypnum cupressiforme* (g,h). The gas concentrations were assessed by gas chromatography. Decrease of N_2O concentration indicates N_2O uptake, increase of CO_2 concentration indicates CO_2 emission.

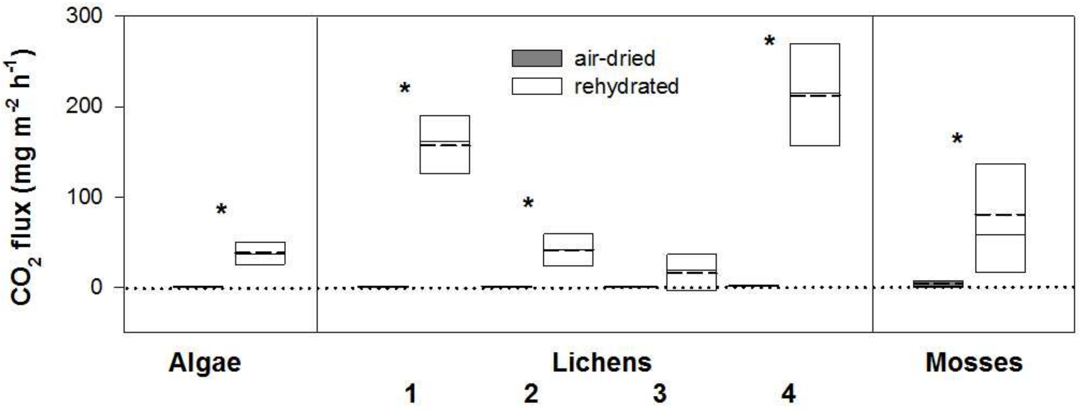

Supplementary Figure S7. CO_2 fluxes in cryptogams sampled from beech bark in Conventwald, Black Forest. Fluxes are expressed per unit of stem projected area. For further information regarding flux determination, taxonomic composition of cryptogams and box plot description, see Fig. 2.

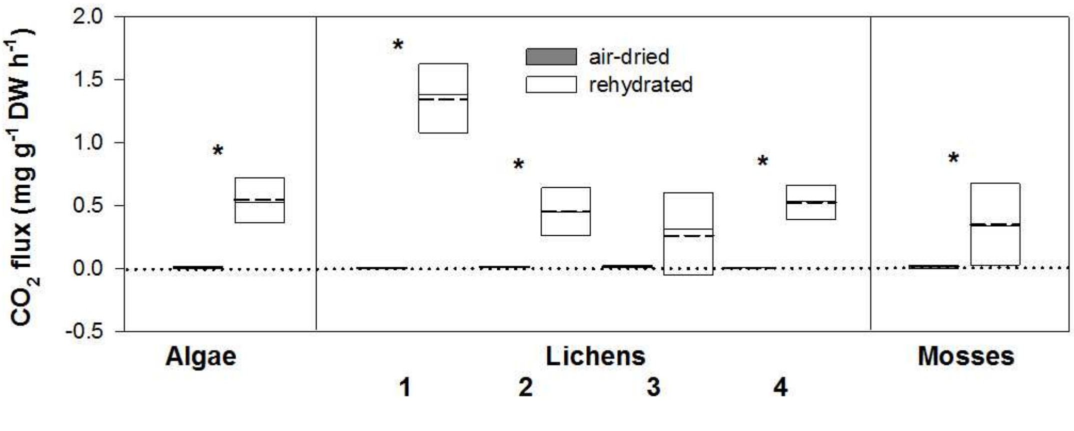

Supplementary Figure S8. CO_2 fluxes in cryptogams sampled from beech bark in Conventwald, Black Forest. Fluxes are expressed per dry weight of cryptogam (DW) unit. For further information regarding flux determination, taxonomic composition of cryptogams and box plot description, see Fig. 2.

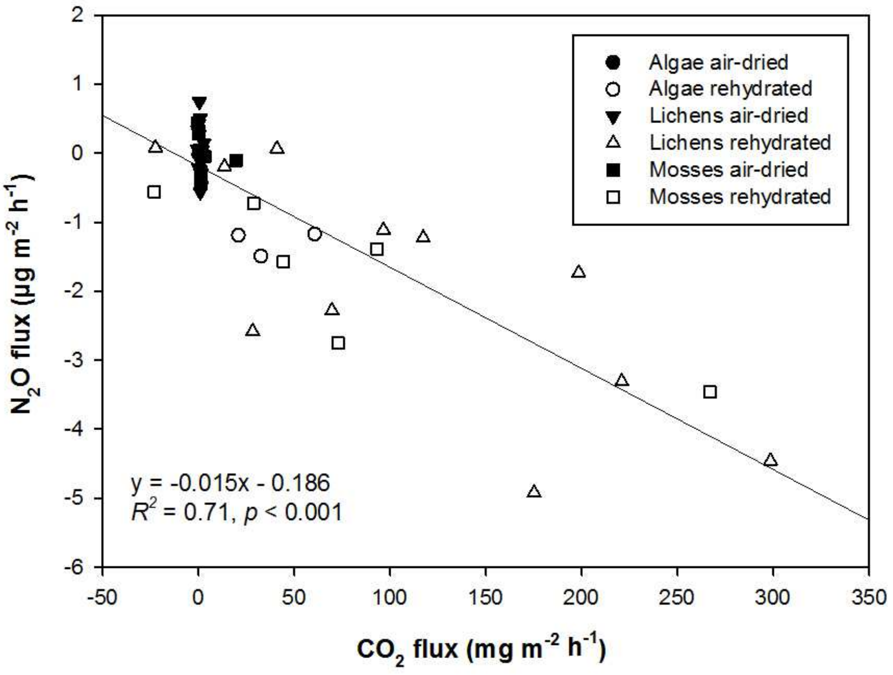

Supplementary Figure S9. N_2O versus CO_2 fluxes in cryptogams sampled from beech bark in Conventwald, Black Forest. For further information regarding flux determination, see Fig. 2.
